# MRI-Based Radiomics Analysis for the Pretreatment Prediction of Pathologic Complete Tumor Response to Neoadjuvant Systemic Therapy in Breast Cancer Patients: A Multicenter Study

**DOI:** 10.3390/cancers13102447

**Published:** 2021-05-18

**Authors:** Renée W. Y. Granzier, Abdalla Ibrahim, Sergey P. Primakov, Sanaz Samiei, Thiemo J. A. van Nijnatten, Maaike de Boer, Esther M. Heuts, Frans-Jan Hulsmans, Avishek Chatterjee, Philippe Lambin, Marc B. I. Lobbes, Henry C. Woodruff, Marjolein L. Smidt

**Affiliations:** 1 Department of Surgery, Maastricht University Medical Center+, P.O. Box 5800, 6202 AZ Maastricht, The Netherlands; snz.samiei@gmail.com (S.S.); e.heuts@mumc.nl (E.M.H.); m.smidt@mumc.nl (M.L.S.); 2 GROW-School for Oncology and Developmental Biology, Maastricht University, P.O. Box 616, 6200 MD Maastricht, The Netherlands; a.ibrahim@maastrichtuniversity.nl (A.I.); s.primakov@maastrichtuniversity.nl (S.P.P.); maaike.de.boer@mumc.nl (M.d.B.); a.chatterjee@maastrichtuniversity.nl (A.C.); philippe.lambin@maastrichtuniversity.nl (P.L.); marc.lobbes@mumc.nl (M.B.I.L.); h.woodruff@maastrichtuniversity.nl (H.C.W.); 3 Department of Radiology and Nuclear Medicine, Maastricht University Medical Center+, P.O. Box 5800, 6202 AZ Maastricht, The Netherlands; thiemo.nijnatten@mumc.nl; 4 The D-Lab, Department of Precision Medicine, Maastricht University, Universiteitssingel 40, 6229 ER Maastricht, The Netherlands; 5 Division of Nuclear Medicine and Oncological Imaging, Department of Medical Physics, University Hospital of Liège and GIGA CRC-In Vivo Imaging, University of Liège, 4000 Liege, Belgium; 6 Department of Nuclear Medicine and Comprehensive Diagnostic Center Aachen (CDCA), University Hospital RWTH Aachen University, 52074 Aachen, Germany; 7 Department of Internal Medicine, Division of Medical Oncology, Maastricht University Medical Center+, P.O. Box 5800, 6202 AZ Maastricht, The Netherlands; 8 Department of Medical Imaging, Zuyderland Medical Center, P.O. Box 5500, 6130 MB Sittard-Geleen, The Netherlands; f.hulsmans@zuyderland.nl

**Keywords:** breast cancer, MRI, neoadjuvant systemic therapy, response prediction, radiomics

## Abstract

**Simple Summary:**

The prediction of pathologic complete response (pCR) to neo-adjuvant systemic therapy (NST) based on radiological assessment of pretreatment MRI exams in breast cancer patients is not possible to date. In this study, we investigated the value of pretreatment MRI-based radiomics analysis for the prediction of pCR to NST. Radiomics, clinical, and combined models were developed and validated based on MRI exams containing 320 tumors collected from two hospitals. The clinical models significantly outperformed the radiomics models for the prediction of pCR to NST and were of similar or better performance than the combined models. This indicates poor performance of the radiomics features and that in these scenarios the radiomic features did not have an added value for the clinical models developed. Due to previous and current work, we tentatively attribute the lack of significant improvement in clinical models following the addition of radiomics features to the effects of variations in acquisition and reconstruction parameters. The lack of reproducibility data meant this effect could not be analyzed. These results indicate the need for reproducibility studies to preselect reproducible features in order to properly assess the potential of radiomics.

**Abstract:**

This retrospective study investigated the value of pretreatment contrast-enhanced Magnetic Resonance Imaging (MRI)-based radiomics for the prediction of pathologic complete tumor response to neoadjuvant systemic therapy in breast cancer patients. A total of 292 breast cancer patients, with 320 tumors, who were treated with neo-adjuvant systemic therapy and underwent a pretreatment MRI exam were enrolled. As the data were collected in two different hospitals with five different MRI scanners and varying acquisition protocols, three different strategies to split training and validation datasets were used. Radiomics, clinical, and combined models were developed using random forest classifiers in each strategy. The analysis of radiomics features had no added value in predicting pathologic complete tumor response to neoadjuvant systemic therapy in breast cancer patients compared with the clinical models, nor did the combined models perform significantly better than the clinical models. Further, the radiomics features selected for the models and their performance differed with and within the different strategies. Due to previous and current work, we tentatively attribute the lack of improvement in clinical models following the addition of radiomics to the effects of variations in acquisition and reconstruction parameters. The lack of reproducibility data (i.e., test-retest or similar) meant that this effect could not be analyzed. These results indicate the need for reproducibility studies to preselect reproducible features in order to properly assess the potential of radiomics.

## 1. Introduction

Neoadjuvant systemic therapy (NST) is increasingly administered in the treatment of breast cancer. The number of breast cancer patients receiving NST varies between 17% and 70% and depends mainly on breast cancer subtype and tumor size [1,2]. NST allows monitoring of in vivo tumor response, potentially decreasing tumor size and thus enabling breast-conserving surgery [1,3,4]. Unfortunately, not all patients respond well to NST, with tumor response ranging from pathologic complete tumor response (pCR) to non-response and sometimes even progression of disease. Predicting which patients will respond well to NST and achieve tumor pCR could lead to modifications of treatment plans. In current clinical practice, magnetic resonance imaging (MRI) assessment combined with clinical (tumor) characteristics is used to determine tumor response to NST [5,6,7]. However, the diagnostic accuracy of the MRI with regard to tumor response evaluation is insufficiently accurate (76.1%) to adapt clinical treatment plans [8]. Furthermore, two studies investigated the use of ultrasound-guided biopsies to identify pCR after NST [9,10]. Unfortunately, the results showed that these biopsies are not accurate enough to identify pCR that surgery can be omitted [11].

Radiomics, a quantitative image analysis technique, could play a role predicting pCR from pretreatment dynamic contrast-enhanced (DCE)-MRI exams. Radiomics extracts large amounts of quantitative features from medical imaging, including MRI. These features capture information on the underlying heterogeneous structure of the region of interest (ROI), describing volume and shape, intensities and textures [12]. Radiomics’ non-invasive ability to characterize the three-dimensional ROI, combined with the availability of ever-growing amounts of (longitudinal) imaging data and its cost-effectiveness, all contribute to the potential use of radiomics in personalized medicine [13,14,15,16]. The emergence of radiomics has so far mainly been applied in the field of clinical oncology and has also permeated breast cancer research.

Several MRI-based radiomics studies have reported promising results regarding the prediction of pCR to NST in breast cancer patients based on pretreatment scans [17,18,19,20,21]. However, the evidence from these studies is limited due to the relatively small sample sizes ranging from 29 to 100 patients and the lack of external validation datasets. Despite the promising potential of radiomics, several hurdles that impede the clinical implementation of radiomics models have been identified. One of these is the sensitivity of radiomics features to the variations in acquisition and reconstruction parameters across different imaging modalities [22,23,24,25,26], and some features were found not to be reproducible even in test-retest scenarios [27,28,29].

This study aimed to investigate the potential of pretreatment contrast-enhanced MRI-based radiomics for the prediction of pCR to NST in breast cancer patients. We hypothesized that radiomics models trained and validated on data from two independent cohorts could add information to the prediction of tumor response to NST and that combined with clinical models can improve prediction accuracy. During our analysis, the sensitivity of radiomics features to the variations in acquisition and reconstruction parameters was established.

## 2. Materials and Methods

### 2.1. Study Population

In this multicenter study, imaging, and clinical data from consecutive women with histopathologically confirmed invasive breast cancer were retrospectively collected from two hospitals in the Netherlands (MUMC+—Maastricht University Medical Center and ZMC—Zuyderland Medical Center) between January 2011 and December 2018. The inclusion criteria were as follows: (i) treated with NST, (ii) have undergone pretreatment DCE-MRI in one of the two participating hospitals, and (iii) breast surgery after NST with histopathological outcome. Exclusion criteria were as follows: (i) histopathologically confirmed inflammatory breast cancer without the possibility of unequivocal tumor segmentation, (ii) MRI exam artefacts, if also rejected for visual assessment by the breast radiologist, (iii) non-standard chemotherapy regimens, deviating from the Dutch breast cancer guidelines, (iv) unfinished NST, and (v) no access to the patient’s medical record. In the case of multifocal breast cancer, all histopathologically confirmed invasive tumors were included in the study. The institutional research board of both hospitals approved the study and waived the requirement for informed consent.

### 2.2. Study Strategy

**Figure 1:**
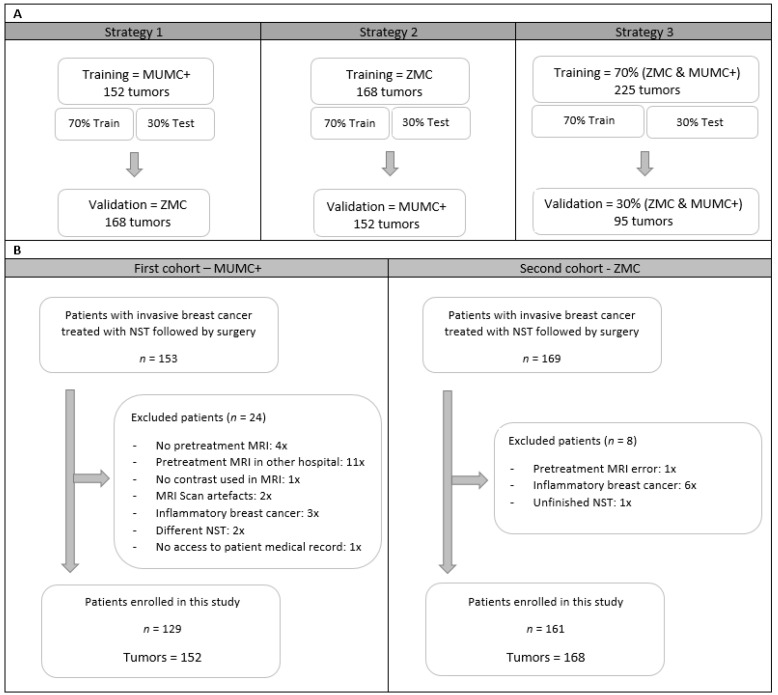
An overview of training, test, and validation data cohorts for the three strategies (**A**) and a flowchart from patient selection for the two different hospitals (**B**). Abbreviations, MUMC+ = Maastricht University Medical Center+, ZMC = Zuyderland Medical Center, NST = Neoadjuvant Systemic Therapy, MRI = Magnetic Resonance Imaging.

As different MRI scanners with varying acquisition and reconstruction parameters were used in the two hospitals, it was decided to develop separate prediction models (radiomics, clinical, and a combination of the two) for both cohorts and to validate them on each other (strategies 1 and 2). Therefore, all feature reduction, selection, and modeling procedures were performed on both data cohorts. A third modelling strategy was based on a mixture of both datasets divided into 70% training and 30% validation cohort. Feature selection and model building was performed on 70% of the training data and tested on the remaining 30% of the training data. The process of splitting the data into training and testing was iterated 100 times, maintaining class imbalance and ensuring that tumors from one patient were selected either in the training data or in the testing data. Figure 1A shows an overview of the selected data per strategy.

### 2.3. Clinical and Pathological Data

Clinical and pathological data were retrieved from patients’ medical records and included age, clinical and pathological tumor, nodes, and metastases (TNM) stage, tumor grade, tumor histology, breast cancer subtype, and NST regimen. The majority of patients were treated with an anthracycline- and taxane-based NST regimen; the remaining received a taxane-based only NST regimen. Human epidermal growth factor receptor 2 (HER2) positive tumors received additional treatment with trastuzumab and/or pertuzumab. After completion of NST, all patients underwent breast surgery. The surgical specimens of all patients were evaluated via standard histopathological analysis by breast pathologists in the two participating hospitals. The breast tumor response was assessed by the Miller–Payne or Pinder grading systems [30,31]. In this study, tumors were defined as pCR when classified as grade 5 using the Miller–Payne classification or classified as 1i and 1ii using the Pinder classification (pCR; ductal carcinoma in situ may be present).

### 2.4. Imaging Data

For all patients, pretreatment MRI exams were collected containing fat-suppressed 3D THRIVE DCE T1-weigthed (T1W), T2-weighted in the MUMC and fat-suppressed T2-weighted in the ZMC, and diffusion weighted imaging sequences. It was decided to only use the peak-enhanced phase of the DCE-T1W images for the radiomics analysis as tumors are best visible on this sequence [32,33]. The DCE-T1W images were obtained before and after intravenous injection of gadolinium-based contrast Gadobutrol (GadovistTM (EU)) with a volume of 15 mL and a flow rate of 2 mL/s. A 105 s temporal resolution protocol was used in the MUMC+ and a 20 s temporal resolution protocol in the ZMC, resulting in five and nineteen post-contrast images for each patient in the MUMC+ and ZMC, respectively. Images were acquired using 1.5T (Ingenia, Intera, and Achieva by Philips Medical system, Best, The Netherlands and Avanto Fit by Siemens, Minhen, Germany) and 3.0T (Skyra by Siemens, Minhen, Germany) MRI scanners. All patients were scanned in prone-position using a dedicated breast-coil. DCE-T1W MRI acquisition protocols from both hospitals can be found in Table 1. Sequence parameters varied per MRI scanner and hospital, reflecting the heterogeneity in medical images used in daily clinical practice.

### 2.5. Tumor Segmentation

The images acquired at tumor peak enhancement, at approximately two minutes’ post-contrast administration, were used for the 3D ROI segmentation and further radiomics analysis, as tumors are best assessed on these images. All histologically confirmed invasive tumors were segmented manually using Mirada Medical’s DBx 1.2.0.59 (64-bit, Oxford, UK) software by a medical researcher with three years of experience (RG), supervised by a dedicated breast radiologist with 14 years of experience (ML). During segmentation, the radiology reports were accessible, and adjustment of image grayscale was allowed to optimize the visualization of the tumor. To gauge any bias introduced by inter-observer segmentation variability, 129 tumors from 102 patients acquired at MUMC+ were segmented by four observers independently with different degrees of experience in breast MR imaging (RG, ML, resident with three years of MRI experience (TvN), and a medical student with no experience (NV)) [34].

### 2.6. Image Pre-Processing and Feature Selection

Image pre-processing of the two-minute postcontrast-T1W images was performed after tumor segmentation using an in-house developed pipeline and using a widely used proposed pre-processing method by Pyradiomics [35,36]. The in-house developed pipeline started first by applying bias field correction to every image using MIM software (version 6.9.4, Cleveland, OH, USA) to correct for nonuniform grayscale intensities in the MRI caused by field inhomogeneities. Second, in order to minimize acquisition-related radiomics variability, voxel dimensions were standardized across the cohorts to arrive at an isotropic voxel resolution of 1 mm3 by means of cubic interpolation [37]. Third, to homogenize arbitrary MRI units and clip image intensities to a certain range, a histogram matching technique was applied, adjusting the pixel values of the MR image such that its histogram matched that of the target MR image from the training data cohort [38,39,40]. Further gray value filtering was applied to generate MRIs with comparable gray value range and to enhance the contrast of the image using the following filtering parameters: window level (WL: 3050) and window width (WW: 2950). Filtering parameters were found when exploring the images after the histogram matching step. Fourth, to reduce high frequency noise and optimize handling of the image, grayscale values were resampled using a fixed bin width of 24, which reduced both image noise and computation times when extracting radiomics features from the ROI [41]. The pre-processing method proposed by Pyradiomics was applied after images’ bias field correction and consisted of *z*-score normalization, resampling to isotropic voxel resolution of 1 mm3, and image discretization using a bin width of 100 to reach an ideal number of bins between 16 and 128 [12].

For each ROI, 833 features were extracted using the Pyradiomics software (version 3.0). The extracted radiomics features included first-order statistics features (18), shape-based features (14), gray-level co-occurrence matrix features (GLCM) (22), gray-level run length matrix features (GLRLM) (16), gray-level size zone matrix features (GLSZM) (16), neighboring gray tone difference matrix features (NGTDM) (5), and gray-level dependence matrix features (GLDM) (14) from both unfiltered and filtered (eight wavelet decompositions) images.

### 2.7. Feature Selection and Radiomics Model Development

**Figure 2:**
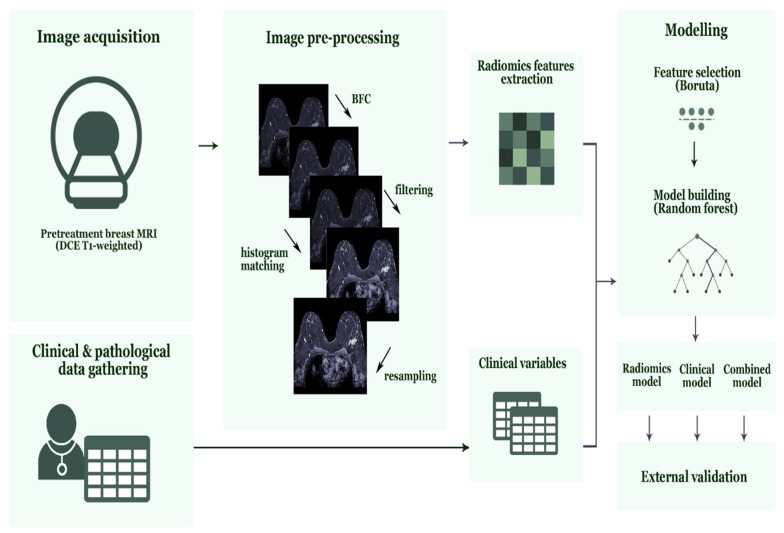
Radiomics workflow used in this study. Abbreviations, MRI = Magnetic Resonance Imaging, DCE = Dynamic Contrast-Enhanced, BFC = Bias Field Correction.

All feature selection steps followed by model development were performed on the 70% training data for each iteration. First, features sensitive to interobserver segmentation variabilities were removed using an intraclass correlation coefficient (ICC) cut-off value >0.75 (29). Consecutively, features with zero or small variance (with the frequency ratio between the most common value and the second most common value larger than 95/5) were removed. This was followed by the removal of highly correlated features using pairwise Spearman correlation (|r| > 0.90), where from any two highly correlated features, the feature with the highest mean correlation with the rest of the features was removed. Finally, the Boruta algorithm, a random forest feature selection method, was used to select important predictive features [42,43]. The Boruta algorithm duplicated all features and shuffled the values in the so-called shadow features. Random forest classifiers were trained on the real and shadow features, and the algorithm subsequently compared the importance score of each feature and selected only those features where the importance of the real feature was higher compared with the shadow’s feature importance [44]. Random forest classification models were trained on the 70% of the training data and tested on the remaining 30% of the training data. The best performing radiomics models according to the summation of AUC and sensitivity value based on the test data in all strategies were selected and validated on the external validation data. All random forest parameters were set at default (Table S1) values. Figure 2 shows the radiomics workflow used in this study. Additionally, the range of the AUC values in the training data set is presented.

### 2.8. Clinical and Combined Model Development

Clinical and combined (based on radiomics features and clinical variables) random forest models were trained, tested, and validated using the same strategy used to develop the radiomics models as described above. Clinical models were based on the available clinical characteristics, including age, clinical tumor stage (cT), clinical nodal stage (cN), clinical tumor grade, tumor histology, and breast cancer subtype. The best performing clinical and combined models according to the summation of AUC and sensitivity value based on the test data in all strategies were selected and validated on the external validation data. All random forest parameters were set as default. Additionally, the range of the AUC values in the training data set was presented.

### 2.9. Statistical Analysis

Image pre-processing steps were performed in Python (version 3.7) using an in-house developed pipeline based on the computer vision packages opencv (version 4.1.0), SimpleITK (version 1.2.0), and numpy (version 1.16.2) procedure. The remaining statistical analysis, feature selection, model development, and model evaluation were performed in R (version 3.6.3) using R studio (version 1.2.1335, Vienna, Austria) [45] and the R packages Boruta (version 7.0.0), Caret (version 6.0–85), Smotefamily (version 1.3.1), RandomForest (version 4.6–14), and pROC, (version 1.3.1) [46]. The difference between cohorts was assessed using independent samples *t*-test for continuous normally distributed variables, and Pearson chi-squared test for categorical variables. Statistical significance was based on *p*-values < 0.05 for both tests. The models developed were evaluated using the AUC and the 95% confidence interval (CI). DeLong’s test was used to compare AUC values. In addition, the sensitivity and specificity and the negative predicted value (NPV) and positive predictive value (PPV) were derived from the confusion matrix. The radiomics quality score (RQS) was used to assess the radiomics workflow [14]. This study checked the Transparent Reporting of a multivariable prediction model for Individual Prognosis or Diagnoses (TRIPOD) guidelines [47,48].

## 3. Results

### 3.1. Patients Demographics

A total of 322 women with invasive breast cancer and treated with NST were considered for inclusion, of whom 32 were excluded (Figure 1B). A total of 290 women with 320 breast tumors met the inclusion criteria, of whom 129 women with 152 breast tumors were collected at the MUMC+ and 161 women with 168 breast tumors at the ZMC. Table 2 summarizes the patient and tumor characteristics of both datasets. The pCR rate of the included tumors was 34.9% (53/152) and 29.2% (49/168) in the MUMC+ and ZMC cohorts, respectively, showing no significant difference. There were significant cohort differences in clinical tumor stage, clinical nodal stage, clinical tumor grade, and tumor histology (Table 3). Clinical tumor stage, clinical tumor grade, and breast cancer subtype showed significant differences between pCR and non-pCR tumors within the individual cohorts (Table 3).

The results reported in the manuscript are based on the in-house developed image preprocessing pipeline, whereas the results based on the image pre-processing proposed by Pyradiomics are reported in the Supplementary Materials (Tables S2 and S3 and Figure S1). In both the radiomics and combined models, no significant differences were found (Table S4).

### 3.2. Radiomics Models—Feature Selection and Model Performance

Of the 833 features extracted per ROI, 87 features were removed, as they were reported to be significantly affected by inter-observer segmentation variability (Table S5). In the best performing radiomics models in all strategies, one feature (*firstorder_maximum*) was removed, as it showed near zero variance. This was followed by the removal of: 574, 568, and 568 highly correlated features in strategy 1, 2, and 3, respectively, leaving 172, 178, and 178 features in the respective cohorts. The Boruta algorithm selected 5, 1, and 6 features in the best performing radiomics models for strategy 1, 2, and 3, respectively (Table 4A).

The results of the best performing radiomics models developed in the three strategies are shown in Table 5A. The AUC values in the validation cohorts were 0.55 (95% CI: 0.46–0.65), 0.52 (95%CI: 0.42–0.62), and 0.50 (95%CI: 0.37–0.64) for the respective strategies 1, 2, and 3. The sensitivity values ranged between 24% and 73% in the validation cohorts. The 100 radiomics models developed in the three strategies resulted in a range of AUC values in the training cohorts between 0.46 and 0.86 (Table S6).

### 3.3. Clinical Models—Feature Selection and Model Performance

**Figure 3:**
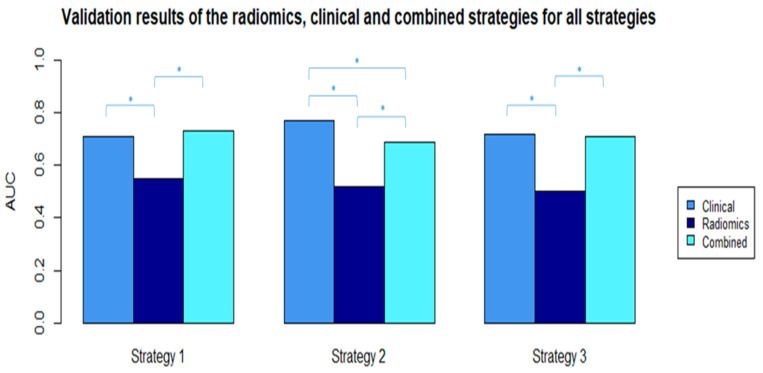
AUC values from the selected radiomics, clinical, and combined validation models in all strategies. * Significant difference between AUC values with *p*-value < 0.05 (*p*-values were calculated using the ROC test by Delong method).

The clinical variables available were patient age, cT, cN, clinical tumor grade, tumor histology, and breast cancer subtype. None of the clinical variables were highly correlated. The Boruta algorithm selected four features in the best performing clinical models for all strategies (Table 4B). The results of the clinical models performed in the three settings are shown in Table 5B. The AUC values in the validation cohorts were 0.71 (95% CI: 0.62–0.79), 0.77 (95% CI: 0.70–0.85), and 0.72 (95% CI: 0.61–0.83) for strategy 1, 2, and 3, respectively. The clinical models performed significantly better compared with the radiomics models (Figure 3). The sensitivity values ranged between 41% and 47% in the validation cohorts. The 100 radiomics models developed in the three strategies resulted in a range of AUC values in the training cohorts between 0.68 and 0.88 (Table S6).

### 3.4. Combined Models—Feature Selection and Model Performance

Of the 833 features extracted per ROI, 87 features were removed, as they were reported to be significantly affected by inter-observer segmentation variability. In the best performing combined models in all strategies, one feature (*firstorder_maximum*) was removed, as it showed near zero variance. This was followed by the removal of 580, 563, and 577 highly correlated features in strategy 1, 2 and 3, respectively, leaving 172, 189, and 175 features in the respective cohorts. The Boruta algorithm selected 7, 4, and 6 features in the best performing radiomics models for strategy 1, 2, and 3, respectively (Table 4C). The three models all contained the same clinical features, clinical tumor grade, and clinical breast cancer subtype. The results of the best performing combined models developed in the three strategies are shown in Table 5C. The AUC values in the validation cohorts were 0.73 (95% CI: 0.65–0.81), 0.69 (95%CI: 0.61–0.78), and 0.71 (95%CI: 0.60–0.81) for the respective strategies 1, 2, and 3. The sensitivity values ranged between 38% and 51% in the validation cohorts. The 100 radiomics models developed in the three strategies resulted in a range of AUC values in the training cohorts between 0.59 and 0.91 (Table S6).

### 3.5. RQS and TRIPOD Results

This study scored a RQS score of 41.7% (15 out of 36 points) (Table S7). The score of the TRIPOD checklist was 73% (24 out of 33 applicable items) (Table S8).

## 4. Discussion

In this multicenter study, we investigated the value of pretreatment contrast-enhanced MRI-based radiomics for the prediction of pCR to NST in breast cancer patients using radiomics, clinical, and combined models in three different data-mixing strategies. The AUC values of the radiomics, clinical, and combined models in the validation datasets of the three strategies had ranges of 0.50–0.55, 0.71–0.77, and 0.69–0.73, respectively. Different radiomics features were selected for the radiomics and combined models in the three strategies, while the selected clinical features were mostly the same in all scenarios, with comparable performances. These results indicate poor performance of the radiomics features and that the radiomic features had no added value to the clinical models developed for the prediction of pCR to NST in breast cancer patients.

The clinical models significantly outperformed the radiomics models for the prediction of pCR to NST in all strategies. This indicates that radiomics features in these scenarios did not have an added value to the clinical model we developed. Furthermore, the variation in the features selected and model performance was greater in the radiomics models compared with the clinical models. However, based on current knowledge in the radiomics field, we cannot say that radiomics features do not have an added value unless the variations in acquisition and reconstruction parameters are properly addressed. Due to the lack of reproducibility data, this study could not analyze the effects of different acquisition and reconstruction parameters on radiomics feature values. Furthermore, the significant differences in population characteristics between the two cohorts could have led to the low performance of the radiomics models. While there was overlap in breast cancer phenotypes, the proportions at which these phenotypes occur may have differed so that the differences in prevalence resulted in differences in overall classification performances.

The results of this study indicate that even extensive MRI pre-processing and homogenization of the MR images do not sufficiently address the variations in acquisition and reconstruction parameters. This is in line with studies published in recent years that investigated the reproducibility of MRI radiomics features in test-retest phantom data as well as in patient data of varying disease sites, and showed that, among others, the variations in acquisition and reconstruction parameters strongly influence the values (concordance) of radiomics features [24,27,28,29,49,50,51,52]. Shur et al. [29] performed a test-retest 1.5T MRI phantom study using the same imaging protocol and showed that 20% of the examined features were not repeatable. A study on repeatability and reproducibility using a T2W pelvic phantom showed that radiomics features values are not only affected by varying acquisition parameters but also by the use of different MRI vendors and magnetic field strengths, wherein the reproducibility of the radiomic features is more affected by difference in MRI vendor than by difference in magnetic field strength [49]. Overall, they reported that only 3.3% (31/944) of the examined features showed excellent robustness (ICC and CCC > 0.9). The radiomics community is currently trying to address these major hurdles.

Investigating comparable published work, we found a number of studies using only univariate predictive features without an external validation data cohort [18,19,20,21,53,54] and more recent published papers that were focusing on multivariate prediction models [32,33,55,56]. Hope Cain et al. [55] achieved an AUC value of 0.71 (95% CI: 0.58–0.83) for predicting pCR to NST in TN/HER2+ breast cancer patients; however, the model was not externally validated. Therefore, we anticipate that the results could not be generalized to scans acquired with different vendors/parameters than those used in the study. The study by Liu et al. [57] was the only study performing external radiomics model validation for the prediction of pCR to NST in breast cancer patients. The study differed from our research by the use of multiparametric (T2-weighted, diffusion-weighted images, and contrast-enhanced T1-weighted) MRI. However, the use of multiple MRI sequences for pCR prediction achieved better outcome with validation AUC values between 0.71 and 0.80. However, it is remarkable that their external validation results were obtained with MRI images that were much less extensively pre-processed compared to our images.

Our study also has its limitations. First, selection bias in retrospective studies is inevitable and so are the biases introduced by clinical protocols, such as HER2+ tumors receiving additional treatment compared to other tumors. Second, since the effect of different MRI scanners and acquisition and reconstruction parameters on radiomics features in breast imaging is not determined, we could not adjust our model for the potential variance induced by these factors in the radiomics feature values. Therefore, since data were collected from two hospitals using five MRI scanners with different acquisition and reconstruction parameters, noise may have been introduced into the models by incorporating radiomics features not robust to these variations. Third, while we believe that MRI preprocessing is a necessary step toward comparable images with intensity values having similar tissue meaning, it is possible that with our choice of preprocessing steps, consistent with current literature, we may have inadvertently removed quantitative information. However, the results obtained with the widely used pre-processing method proposed by Pyradiomics showed no significant differences from the result reported here. Fourth, the number of patients included in this study did not allow us to perform a subanalysis for the different breast cancer subtypes. Fifth, the data were collected over a relatively long period of time during which optimization of MRI acquisitions protocols occurred, which may have introduced variations as well. Last, for these analyses it was specifically chosen to use the peak-enhanced (2 min) post-contrast T1W images, as breast tumors are most visible on them and because some of the tumors included cannot be seen on other sequences; for example, mucinous tumors and some of the invasive lobular tumors are not or only weakly visible on the subtraction images. In our opinion, performing the analysis using the subtraction images instead of the peak-enhanced images would have resulted in a significant decrement in the number of patients that could be analyzed. Furthermore, as the effects of the different breast MRI sequences on the radiomics features is not yet understood, future radiomics research in the field of breast cancer could focus on the use of the different MRI sequences, as well as on multiparametric and delta radiomics approaches.

## 5. Conclusions

In conclusion, this study showed no contribution of pretreatment contrast-enhanced MRI-based radiomics for the prediction of tumor pCR on NST in breast cancer patients, as neither the radiomics nor the combined models performed significantly better than the clinical models. However, without analysis of the effects of variations in acquisition and reconstruction parameters, it is currently not possible to conclude that pretreatment contrast-enhanced MRI-based radiomic features have no value in the prediction of pCR to NST. The effects of different acquisition and reconstruction parameters on radiomics feature values in breast imaging should be explored in future MRI-breast reproducibility studies to investigate whether further research into pretreatment MRI-based radiomics for the prediction of pCR to NST in breast cancer patients is useful.

## Figures and Tables

**Table 1:** Scanning Parameters.

Hospital	Scanner	Total MRI Exam No.	Group	No. of Tumors for Specific Scanning Parameters	Pixel Spacing	Acquisition Matrix (*n*)	Slice Thickness (mm)	TR/TE (ms) (*n*)	Spacing between Slices	Flip Angle
MUMC+	Philips 1.5T (Ingenia)	124	a	44	(0.97, 0.97)	340 × 340	1	3.4/7.5 3.5/7.6	1	10°
			b	66	(0.95, 0.95)	378 × 314 (28) 380 × 318 (23) 380 × 316 (18)	1	3.2/7.1 3.4/7.5 3.5/7.6	1	10°
			c	9	(0.80, 0.80)	344 × 344	1	3.4/7.5	1	10°
			d	3	(0.92, 0.92)	400 × 333 (2) 398 × 331 (1)	1	3.5/7.6 3.4/7.5	1	10°
			e	1	(0.88, 0.88)	384 × 368	1	3.4/7.5	1	10°
			f	1	(0.85, 0.85)	384 × 278	1	2.9/6.5	1	10°
	Philips 1.5T (Intera)	28	a	25	(0.97, 0.97)	340 × 337	1	3.4/7.4-7.6	1	10°
			b	1	(0.99, 0.99)	376 × 376	1	3.4/7.4	1	10°
			c	1	(0.95, 0.95)	364 × 364	1	3.4/7.5	1	10°
			d	1	(0.85, 0.85)	368 × 368	1	3.4/7.4	1	10°
ZMC	Philips 1.5T (Achieva)	123	a	94	(0.97, 0.97)	340 × 338	2	3.4/6.9–7.0	1	12°
			b	28	(0.96, 0.96)	372 × 368 (15) 372 × 370 (13)	2	3.4/6.9–7.0	1	12°
			c	1	(0.90, 0.90)	392 × 388	2	3.4/6.9	1	12°
	Siemens 3.0T (Skyra)	39	a	39	(0.69, 0.69)	288 × 288	2	1.2/4.0	unknown	10°
	Siemens 1.5T (Avanto_fit)	6	a	6	(0.89, 0.89)	224 × 202	2	2.4/6.1	unknown	10°

Abbreviations, MRI = Magnetic Resonance Imaging, TR = Repetition Time, TE = Echo Time, T = Tesla, MUMC+ = Maastricht University Medical Center+, ZMC = Zuyderland Medical Center.

**Table 2:** Clinical patient and tumor characteristics of patients in both complete data from the Maastricht University Medical Center+ (MUMC+) and Zuyderland Medical Center (ZMC) hospital.

Characteristics	MUMC+	ZMC	*p*-Value
Number of patients	129	161	-
Patient Age (years) (mean; range)	51 (28–73)	52 (28–79)	0.378
Number of tumors	152	168	-
Clinical tumor stage (%)			0.007
T1	29 (19.1)	16 (9.5)	
T2	99 (65.1)	103 (61.3)	
T3	20 (13.2)	37 (22.0)	
T4	4 (2.6)	12 (7.2)	
Clinical nodal stage (%)			<0.001
N0	88 (57.9)	59 (35.1)	
N1	44 (29.0)	87 (51.8)	
N2	9 (5.9)	12 (7.1)	
N3	11 (7.2)	7 (4.2)	
Unknown	0 (0.0)	3 (1.8)	
Clinical tumor grade (%)			0.003
1	8 (5.3)	22 (13.1)	
2	70 (46.1)	84 (50.0)	
3	68 (44.7)	62 (36.9)	
Unknown	6 (3.9)	0 (0.0)	
Tumor histology (%)			0.009
Invasive ductal carcinoma	136 (89.5)	134 (79.8)	
Invasive lobular carcinoma	10 (6.6)	14 (8.3)	
Invasive mixed ductal/lobular carcinoma	0 (0.0)	9 (5.4)	
Other invasive carcinoma	6 (3.9)	11 (6.5)	
Cancer Subtype (%)			0.921
HR+ and HER2−	80 (52.6)	82 (48.8)	
HR+ and HER2+	22 (14.5)	26 (15.5)	
HR− and HER2+	19 (12.5)	22 (13.1)	
Triple-negative	31 (20.4)	38 (22.6)	
Response to NAC (%)			0.331
pCR	53 (34.9)	49 (29.2)	
Non-pCR	99 (65.1)	119 (70.8)	

Abbreviations, HR = Hormone Receptor, HER2 = Human Epidermal growth factor Receptor 2.

**Table 3:** Clinical patient and tumor characteristics of patients in both complete data cohorts on pCR and non-pCR tumors from the Maastricht University Medical Center (MUMC+) and Zuyderland Medical Center (ZMC) hospitals.

Characteristics	MUMC+			ZMC		
	Non-pCR	pCR	*p*-Value	Non-pCR	pCR	*p*-Value
Number of tumors	99	53	-	119	49	-
Patient Age (years) (mean; range)	52 (32–72)	51 (28–73)	0.600	53 (31–79)	52 (28–73)	0.538
Clinical tumor stage (%)			0.019 *			0.023
T1	12 (12.1)	17 (32.1)		6 (5.0)	10 (20.4)	
T2	68 (68.7)	31 (58.5)		76 (63.9)	27 (55.1)	
T3	16 (16.2)	4 (7.5)		28 (23.5)	9 (18.4)	
T4	3 (3.0)	1 (1.9)		9 (7.6)	3 (6.1)	
Clinical nodal stage (%)			0.943			0.526
N0	56 (56.6)	32 (60.3)		39 (32.8)	20 (40.8)	
N1	29 (29.3)	15 (28.3)		62 (52.1)	25 (51.0)	
N2	6 (6.1)	3 (5.7)		11 (9.2)	1 (2.0)	
N3	8 (8.1)	3 (5.7)		5 (4.2)	2 (4.1)	
Unknown	0 (0.0)	0 (0.0)		2 (1.7)	1 (2.0)	
Clinical tumor grade (%)			<0.001 *			0.002
1	8 (8.1)	0 (0.0)		19 (15.9)	3 (6.1)	
2	58 (58.6)	12 (22.7)		66 (55.5)	18 (36.7)	
3	32 (32.3)	36 (67.9)		34 (28.6)	28 (57.2)	
Unknown	1 (1.0)	5 (9.4)		0 (0.0)	0 (0.0)	
Tumor histology (%)			0.913			0.030
Invasive ductal carcinoma	89 (89.9)	47 (88.7)		91 (76.5)	43 (87.8)	
Invasive lobular carcinoma	6 (6.1)	4 (7.5)		13 (10.9)	1 (2.0)	
Invasive mixed ductal/lobular carcinoma	0 (0.0)	0 (0.0)		9 (7.6)	0 (0.0)	
Other invasive carcinoma	4 (4.0)	2 (3.8)		6 (5.0)	5 (10.2)	
Cancer Subtype (%)			<0.001 *			<0.001
HR+ and HER2−	64 (64.6)	16 (30.2)		75 (63.0)	7 (14.3)	
HR+ and HER2+	15 (15.2)	7 (13.2)		14 (11.8)	12 (24.5)	
HR− and HER2+	6 (6.1)	13 (24.5)		5 (4.2)	17 (34.7)	
Triple-negative	14 (14.1)	17 (32.1)		25 (21.0)	13 (26.5)	

Abbreviations, pCR = pathologic Complete Response, HR = Hormone Receptor, HER2 = Human Epidermal growth factor Receptor 2.

**Table 4:** Selected features in best performing radiomics, clinical, and combined models for the three strategies.

	Strategy 1	Strategy 2	Strategy 3
**A (Radiomics)**	O_glszm_GrayLevelVariance	W.LHH_firstorder_Kurtosis	O_shape_Sphericity
	W.HLL_firstorder_Mean		W.LLH_glszm_GrayLevelNon- Uniformity
	W.HLL_glcm_Imc1		W.LLH_glszm_ZoneEntropy
	W.HLH_glcm_InverseVariance		W.HHL_glcm_Imc1
	W.LLL_ngtdm_Complexity		W.HHH_glrlm_RunEntropy
			W.LLL_glcm_DifferenceVariance
**B (Clinical)**	Age	cT	Age
	cT	cN	cT
	Tumor grade	Tumor grade	Tumor grade
	Breast cancer subtype	Breast cancer subtype	Breast cancer subtype
**C (Combined)**	Tumor grade	Tumor grade	cT
	Breast cancer subtype	Breast cancer subtype	Tumor grade
	O_shape_Sphericity	W.LHL_firstorder_kurtosis	Breast cancer subtype
	O_firstorder_Mean	W.HHL_gldm_DependenceVariance	O_shape_Sphericity
	W.HLL_glcm_Imc2		W.LLH_glszm _SmallAreaLowGrayLevelEmphasis
	W.HLL_glszm_ZoneEntropy		
	W.HLH_glcm_InverseVariance		

Abbreviations: O = original, W = wavelet, cT = clinical tumor stage, and cN = clinical nodal stage.

**Table 5:** Performance of best performing random forest radiomics (5A), clinical (5B), and combined (5C) models for the three strategies.

**A (Radiomics)**	**Strategy 1**			**Strategy 2**			**Strategy 3**		
	**Training** **MUMC+**		**Validation** **ZMC**	**Training** **ZMC**		**Validation** **MUMC+**	**Training** **70% Mixed**		**Validation** **30% Mixed**
	**Train**	**Test**		**Train**	**Test**		**Train**	**Test**	
Area under the ROC	0.71	0.78	0.55	0.64	0.67	0.52	0.60	0.65	0.50
95% CI	0.59–0.82	0.63–0.92	0.46–0.65	0.54–0.75	0.49–0.84	0.42–0.62	0.49–0.71	0.51–0.80	0.37–0.64
Sensitivity (%)	53	59	73	44	60	28	38	48	24
Specificity (%)	89	79	36	75	72	62	92	77	88
PPV (%)	70	63	32	42	47	28	69	48	47
NPV (%)	79	76	77	77	81	62	75	77	72
**B (Clinical)**	**Strategy 1**			**Strategy 2**			**Strategy 3**		
	**Training** **MUMC+**		**Validation** **ZMC**	**Training** **ZMC**		**Validation** **MUMC+**	**Training** **70% Mixed**		**Validation** **30% Mixed**
	**Train**	**Test**		**Train**	**Test**		**Train**	**Test**	
Area under the ROC	0.79	0.81	0.71	0.81	0.84	0.77	0.75	0.86	0.72
95% CI	0.71–0.87	0.68–0.95	0.62–0.79	0.73–0.89	0.72–0.96	0.70–0.85	0.68–0.83	0.77–0.95	0.61–0.83
Sensitivity (%)	54	86	45	54	71	47	52	71	41
Specificity (%)	87	64	74	85	86	85	77	84	78
PPV (%)	69	57	42	59	67	63	52	68	46
NPV (%)	78	89	77	82	88	75	77	86	75
**C (Combined)**	**Strategy 1**			**Strategy 2**			**Strategy 3**		
	**Training** **MUMC+**		**Validation** **ZMC**	**Training** **ZMC**		**Validation** **MUMC+**	**Training** **70% Mixed**		**Validation** **30% Mixed**
	**Train**	**Test**		**Train**	**Test**		**Train**	**Test**	
Area under the ROC	0.82	0.83	0.73	0.79	0.86	0.69	0.79	0.86	0.71
95% CI	0.74–0.90	0.70–0.97	0.65–0.81	0.71–0.88	0.74–0.98	0.61–0.78	0.73–0.86	0.76–0.96	0.60–0.81
Sensitivity (%)	53	67	51	51	71	51	52	71	38
Specificity (%)	88	88	82	87	82	67	85	89	83
PPV (%)	69	77	53	62	63	45	61	75	50
NPV (%)	78	82	80	81	88	72	79	87	75

Abbreviations, MUMC+ = Maastricht University Medical Center+, ZMC = Zuyderland Medical Center, CI = confidence interval, PPV = positive predicted value, NPV = negative predicted value.

## Data Availability

The data presented in this study are available on reasonable request from the corresponding author. Due to privacy restrictions the data are not publicly available.

## Supplementary Materials

The following are available online at https://www.mdpi.com/article/10.3390/cancers13102447/s1, Supplementary Materials include Table S1: Default random forest parameters, Table S2: Selected features using the proposed image pre-processing method by Pyradiomics, Table S3: Results of the best performing models using the proposed image pre-processing method by Pyradiomics, Figure S1: Comparisons of the validation AUC values using the proposed image pre-processing method by Pyradiomics, Table S4: Comparison of the validation results of both pre-processing methods, Table S5: List of excluded features affected by inter-observer segmentation variability, Table S6: Ranked AUC values of the 100 radiomics, clinical, and combined trainings models for the three strategies, Table S7: Radiomics Quality Score, and Table S8: TRIPOD Checklist.
